# Building vs. Rebuilding Epidermis: Comparison Embryonic Development and Adult Wound Repair

**DOI:** 10.3389/fcell.2021.796080

**Published:** 2022-01-25

**Authors:** Sangbum Park

**Affiliations:** ^1^ Institute for Quantitative Health Science and Engineering (IQ), Michigan State University, East Lansing, MI, United States; ^2^ Division of Dermatology, Department of Medicine, College of Human Medicine, Michigan State University, East Lansing, MI, United States; ^3^ Department of Pharmacology and Toxicology, College of Human Medicine, Michigan State University, East Lansing, MI, United States

**Keywords:** wound repair, development, skin, epidermis, tissue-resident immunity

## Abstract

Wound repair is essential to restore tissue function through the rebuilding of pre-existing structures. The repair process involves the re-formation of tissue, which was originally generated by embryonic development, with as similar a structure as possible. Therefore, these two processes share many similarities in terms of creating tissue architecture. However, fundamental differences still exist, such as differences in the cellular components, the status of neighboring tissues, and the surrounding environment. Recent advances in single-cell transcriptomics, *in vivo* lineage tracing, and intravital imaging revealed subpopulations, long-term cell fates, and dynamic cellular behaviors in live animals that were not detectable previously. This review highlights similarities and differences between adult wound repair and embryonic tissue development with a particular emphasis on the epidermis of the skin.

## Introduction

The epidermis is the topmost layer of the skin. It plays an important role in protecting our bodies from the surrounding environment such as temperature, pH extremes, pathogens, UV, and mechanical stress. The skin epithelium builds a rigid structure by forming cellular junctions between cells such as desmosomes, adherens, and tight junctions. While stratified epithelium acts as physical barriers, tissue-resident immune cells perform immunological barrier functions. These immune cells surveil the surface of the skin and protect our body from diverse insults such as pathogens and tumors by mediating immunity and tolerance. The epidermis is an ideal model system to investigate the repair process because its position as the outermost protective layer of the body means it is highly prone to injury. This review will discuss how the epidermal barrier is built during development and how the rebuilding process restores the damaged barrier after injury. In addition, direct comparison of the same cellular components during these two distinct processes will provide broad insights into epidermal regeneration.

### Physical and Immunological Barriers of the Epidermis

**FIGURE 1:**
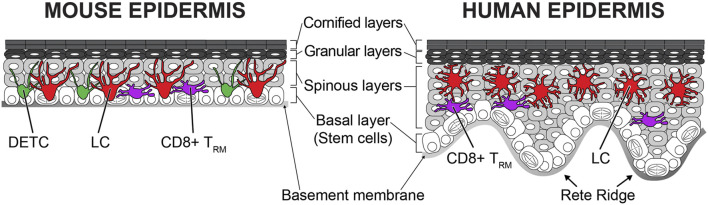
Structure and cellular components of mouse and human epidermis. Highly organized epithelial cells composed of stratified epithelium in the epidermis. The bottommost basal layer cells are attached to the basement membrane. Epithelial stem cells are in the basal layer and differentiate upward. Both mouse and human epidermis have four different layers (basal, spinous, granular, and cornified layers). The human epidermis is much thicker than the mouse epidermis and has an undulated rete ridge structure. Both undifferentiated and differentiated epithelial cells form cellular junctions to build the solid stratified structure. The stratified epithelium acts as physical barriers from outer environment. There are tissue-resident immune cells within the epidermis. Langerhans cells (LCs), dendritic epidermal T cells (DETCs), and CD8^+^ tissue-resident memory T cells (T_RM_s) exist in the mouse epidermis, but humans do not have the DETCs. These immune cells continuously surveil possible infections and perform immunological barrier functions.

The skin epithelium has a highly organized structure (Figure 1). Undifferentiated epithelial cells are located at the bottommost basal layer (also known as stratum basale). The basal layer cells gradually and continuously differentiate from bottom to top. Basal layer cells are anchored to the basement membrane *via* hemidesmosomes and focal adhesions. Only basal epithelial cells can self-renew during homeostasis postnatally. The human epidermis has a rete ridge structure because the uneven epidermal thickness is undulated. Depending on the spatial distribution, basal epithelial cells show different characteristics. Cells located at the bottom of the rete ridge express high levels of integrin *α*6 and kertin15, and cells at the top of the rete ridge express high levels of integrin β1, MCSP, and Lrig1 (Jones et al., 1995; Li et al., 1998; Legg et al., 2003; Webb et al., 2004; Jensen and Watt, 2006). It remains controversial which cells have higher stemness, but a transplantation study suggested that some cells have the capacity for long-term self-renewal. Transplantation of human epidermis in a patient with junctional epidermolysis bullosa showed that there are a limited number of long-lived stem cells. These stem cells have the potency to self-renew in the long term and to generate progeny (Hirsch et al., 2017). As aging progresses, the skin epidermis loses the rete ridge structure and becomes flat (Giangreco et al., 2010). Similar to aged human epidermis, mouse skin does not have rete ridges and remains flat throughout life. There are several hypotheses about stem cells in the basal layer in the mouse epidermis. Conclusions differ depending on the experimental designs (lineage tracing with different mouse lines, label-retention, and intravital imaging) and region of skin (back, tail, ear, and paw) (Gonzales and Fuchs, 2017). The label retention approach with the H2B-GFP pulse-chase system exhibited stem cells spatially organized depending on the position of scale/interscale regions and blood vessels in the mouse back and tail epidermis (Gomez et al., 2013; Sada et al., 2016). Lineage tracing by Cre recombinase [keratin 14 promoter (K14)-CreER, involucrin promoter (Inv)-CreER] demonstrated that quiescent stem cells are stochastically positioned in the epidermis of the mouse tail skin and that these cells contribute the long-term homeostasis and wound repair (Mascré et al., 2012). However, lineage tracing with a different CreER marker [Cyp1a1 promoter (Ah)-CreER] in the tail skin supports a stochastic model (Clayton et al., 2007). Intravital imaging from the ear and paw also showed that all basal epithelial cells equally possess the potency to self-renew and differentiate with random fate decisions (stochastic) (Doupé et al., 2010; Rompolas et al., 2016). Additional studies are needed to elucidate the stemness of basal epithelial cells in the epidermis from both humans and mice. Since mouse skin is more experimentally tractable, the rest of this review will summarize what is known about the development and the wound repair from mouse studies.

Basal epithelial cells differentiate by delaminating from the basement membrane and moving sequentially through the upper spinous, granular, and cornified layers. Differentiating cells change not only position but also cellular characteristics. During differentiation, basal epithelial cells stop expressing the integrins that form focal adhesions and hemidesmosomes (Fuchs and Raghavan, 2002). The expression of P-cadherin also disappears, but E-cadherin is continuously maintained to form adherens junctions. Adhesion *via* desmosomes is also sustained, but isotypes of the desmocollins and desmoglein, which constitute the desmosomes, are gradually changed (Simpson et al., 2011). Microtubule organization also changes during differentiation. Microtubules are rearranged from a centrosomal array in the basal layer to cortical localization in the suprabasal layers. The microtubule rearrangement during the differentiation might be related to the inactivation of the centrosome which blocks cell cycle progression of suprabasal cells (Lechler and Fuchs, 2007; Muroyama and Lechler, 2017). Basal epithelial cells express keratins 5 and 14, which are gradually replaced with keratins 1 and 10 during differentiation (Simpson et al., 2011). It was reported that keratin 10 expression is already detectable in cells located in the basal layer (Schweizer et al., 1984). More recently, several studies expanded on this finding by showing more globally that the transcriptional program associated with differentiation (including keratin 10 but also other things like Desmoglein 1, suprabasin, and Krtdap) (Haensel et al., 2020; Wang et al., 2020; Cockburn et al., 2021). This suggests that the differentiation process is initiated in the basal layer before delamination occurs. The spinous layers (also known as stratum spinosum) are the layers following the basal layer. The spinous layers provide a strong barrier function by establishing a strong intercellular connection and thick keratin bundles (Fuchs, 2009). The granular layers (also known as stratum granulosum) are the upper layers of the spinous layers. Cells in the granular layers form intercellular tight junctions which prevent penetration of outer pathogens and body water loss (Fuchs, 2008). The granular layer cells have keratohyalin granules within as their nomenclatures suggest (Goleva et al., 2019). The granules are membraneless protein deposits, and their function is still unclear. Recently, it was revealed that filaggrin assembles keratohyalin granules *via* liquid–liquid phase separation (Quiroz et al., 2020). The cornified layers (stratum corneum) are the outmost layers of the epidermis. These are dead cells that are composed mainly of keratin and cell membrane. There are lipids composed of ceramides, cholesterol, and fatty acids between cells (Lippens et al., 2005). The cornified cells and intercellular lipids protect our skin by regulating water loss, acidic pH, permeability, and skin microbiome (Del Rosso and Levin, 2011).

There are three epidermis resident immune cells, Langerhans cells (LCs), dendritic epidermal T cells (DETCs), and CD8^+^ tissue-resident memory T cells (T_RM_s) (Chong et al., 2013). These immune cells form the first-line barrier from the environment and work as sentinels by surveilling the surface and neighboring cells of the epidermis (Figure 1). LCs are well-known antigen-presenting cells. LCs express tight junction proteins and extend their dendrites upward to interact with gap junctions in the granular layer (Kaplan, 2017). Mature LCs can penetrate tight junctions with the tip of their dendrites and capture antigens on the skin surface (Kubo et al., 2009; Ouchi et al., 2011). LCs become activated after antigen uptake. The expression of MHC-II, CD80, and CD86 is increased in activated LCs (Rattis et al., 1996). Although LCs are static during homeostasis, activated LCs gain high mobility. Activated LCs mobilize by disconnecting from epithelial cells by decreasing E-cadherin and EpCam expression (Brand et al., 2019). Interestingly, the inhibition of E-cadherin or EpCAM in LCs does not promote the exit of LCs from the epidermis (Ouchi et al., 2016; Brand et al., 2019). These data suggest that adhesion molecules do not directly regulate the migration of LCs in the absence of inflammation. Activated LCs migrate to the lymphatic vessels by using C-X-C motif chemokine receptor 4 (CXCR4) and CC chemokine receptor 7 (CCR7) and enter the lymphatic system (Ohl et al., 2004; Ouwehand et al., 2008). Eventually, these cells migrate into the lymph nodes to present antigen to naive T cells (Otsuka et al., 2018).

The dendritic epidermal T cells (DETCs) are *γδ* T cells in the epidermis. These cells exist only in the mouse epidermis but not in the human epidermis (Nielsen et al., 2017). The majority of T cells have classical *αβ* T-cell receptors (TCRs), whereas DETCs have *γδ* TCRs similar to intestinal intraepithelial lymphocytes (IELs) (Vandereyken et al., 2020). The *γδ* T cells are classified as innate-like lymphocytes with the NK cells. Therefore, DETCs have characteristics from both innate and adaptive immunities (Bennett et al., 2015). DETCs show innate pattern recognition and NKG2D ligand expression, like innate immune cells, and express a rearranged TCR, like adaptive immune cells (MacLeod and Havran, 2011). DETCs have a dendritic morphology and the tip of their dendrites contact tight junctions and surveil the outer environment like LCs (Chodaczek et al., 2012). Although TCR ligands of DETCs are still unknown, there are several pieces of evidence indicating that DETCs react to bacterial infection, tumor cells, and stress signals from neighboring epithelial cells (Hayday, 2009; MacLeod and Havran, 2011; Komori et al., 2012; Vermijlen et al., 2018). Unlike dynamic IELs, DETCs are more static, similar to LCs during homeostasis (Hoytema van Konijnenburg et al., 2017). The difference in the behaviors of these two *γδ* T-cell types could be dictated by the structural and environmental differences between the skin and the intestine. The skin is composed of stratified epithelium with a dry surface, whereas the intestine is a simple squamous epithelium and has many nutrients on the surface which favors the microbiome (Grice and Segre, 2011; Byrd et al., 2018; Fan and Pedersen, 2021). Recently, it has been shown that LCs and DETCs actively maintain their regular distribution during homeostasis (Park et al., 2021). Remarkably, these immune cells recover regular distribution within a few days after local perturbation with laser ablation and after global depletion with diphtheria toxin-induced cell death. It is revealed that dendritic interaction is important for maintaining a regular distribution, but the underlying mechanisms remain unclear. Interestingly, depletions of one immune population do not impact the other’s patterning (Park et al., 2021). These data suggest that although LCs and DETCs have a similar tiling pattern, these cells maintain the pattern independently through different mechanisms.

Immunological memory is a key factor of adaptive immunity for long-term protection. The skin also has memory function from tissue-resident memory T (T_RM_) cells (Hirai et al., 2021). In the epidermis, CD8^+^ T_RM_ cells are predominant, whereas CD4^+^ T_RM_ cells mainly exist in the dermis (Szabo et al., 2019). After infection, CD8^+^ T_RM_ cells repopulate the infected epidermis and are maintained by IL-7, IL-15, and TGF-*β* (Ariotti et al., 2012; Adachi et al., 2015; Dijkgraaf et al., 2019). These cells have a memory to a specific antigen (Hirai et al., 2021). After antigen recognition, these cells massively proliferate and differentiate into cytotoxic effector T cells (Menares et al., 2019). In contrast to LCs and DETCs, CD8^+^ T_RM_ cells are mobile within the epidermis during homeostasis (Ariotti et al., 2012; Zaid et al., 2014). The higher mobility enables CD8^+^ T_RM_ cells to cover the epidermis with lower density than LCs and DETCs. Interestingly, a territory of CD8^+^ T_RM_ cells can overlap with LCs but not with DETCs (Zaid et al., 2014). This suggests that CD8^+^ T_RM_ cells and DETCs have a repulsive interaction. This exclusion could indicate functional redundancy between T cells.

### Formation and Repair of the Skin Epithelium

**FIGURE 2:**
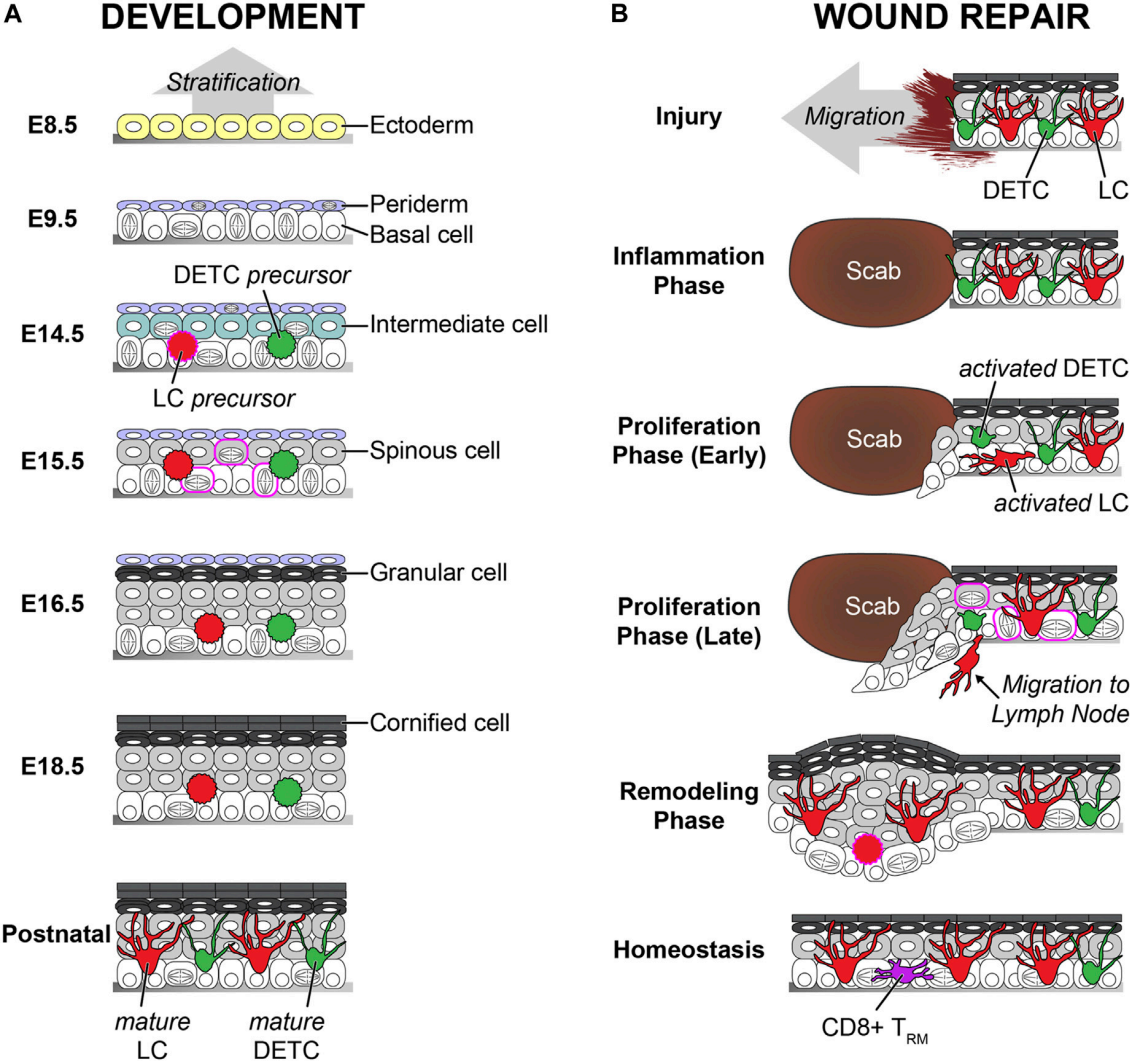
Comparison of the development and the wound repair of the epidermis. **(A)** The vertical stratification builds the epidermis during the development. Ectoderm-derived single layer cells on the basement membrane differentiate to the basal layer cells. Basal layer cells make the periderm on the top and the intermediated layer between the basal layer and the periderm. The intermediate cells change to spinous cells. The spinous cells differentiate into granular cells. The periderm sheds off at the later stage of the embryogenesis, and the granular cells differentiate to the cornified cells before birth. Both precursors from LCs and DETCs are seeded in the epidermis at the early embryogenesis. These immature cells increase the number by proliferation burst and change to dendritic morphology by maturation after birth. The embryonic days (E) are based on mouse embryogenesis. **(B)** The lateral migration rebuilds the damaged epidermis during the wound repair. The inflammation phase is initiated right after the injury. Next, the re-epithelialization starts during the proliferation phase. Both undifferentiated basal and differentiated spinous cells migrate together. Proliferation is dramatically increased. Migrating cells and cells in the spinous layer show cell division during re-epithelialization, which are not observed during homeostasis. The epidermis becomes thick after the re-epithelialization, and the thickness gradually decreases during the remodeling phase. Both DETCs and LCs are activated after the inflammation phase. The activated DETCs become round, and the activated LCs lose contact with the tight junctions. The activated LCs leave the epidermis to migrate to the lymph node. LC density is low right after the re-epithelialization, but LCs recover the normal density during the remodeling phase. The DETCs do not exist in the neo-epidermis, and CD8^+^ TRMs replace the territory of the DETCs. Similar features between the development and the wound repair are labeled in magenta. During the wound repair, the proliferation rate is dramatically increased, and even asymmetric and spinous divisions are observed like the development. In addition, there is a possibility that LC precursors repopulate on the neo-epidermis like the development.

During embryogenesis, skin epidermis originates from the ectoderm (Koster and Roop, 2007). A single layer of surface ectoderm, which expresses keratins 8 and 18, is formed on the basement membrane. These cells differentiate to keratins 5 and 14 positive epidermal lineage cells by BMP and p63 signaling (Lutz et al., 2010). p63 knockout studies revealed that p63 is a master regulator of epidermal stratification (Mills et al., 1999; Yang et al., 1999). An additional layer, called periderm, is also formed above the basal layer at this time point. The periderm layer is transient during embryogenesis and acts as a protective barrier by preventing immature adhesion between epithelial cells during development (Richardson et al., 2014). Asymmetric cell divisions of basal cells make an intermediate layer. Cells in the intermediate layer become the spinous layer (Lechler and Fuchs, 2005). Differentiation of spinous layer cells creates a granular layer above the spinous layer. The periderm layer sheds off in the late stage of embryogenesis, and the cornified layers are formed on the top. To build the fully differentiated epidermis by using a 3D *ex vivo* skin organ culture system, keratinocytes need to be incubated at the air–liquid interface (Carlson et al., 2008). In contrast, full-term neonates have a fully developed epidermis with the cornified layers, despite the epidermis being exposed to amniotic fluid (Oranges et al., 2015) (Figure 2).

Wound repair in adult skin is a complex process in which diverse cell types participate. Generally, it is divided into three phases: inflammation, proliferation, and remodeling (Gurtner et al., 2008). First, the inflammation phase starts immediately after the injury. Immune cells, such as neutrophils and macrophages, are recruited at the site of injury and clean up dead cells and bacteria. Next, the proliferation stage follows inflammation as cells in the skin reconstitute the damaged structure of the skin, including epithelium, extracellular matrix, and blood vessels. After skin injury, adjacent epithelial cells seal the damaged area *via* re-epithelialization, also known as epidermal repair. Abnormal re-epithelialization enhances the opportunity for additional infections which lead to the development of severe diseases, such as non-healing chronic wounds (Eming et al., 2014). Whereas the single basal layer forms stratified epithelium *via* vertical differentiation during development, and the damaged epidermis is restored by the lateral movement of adjacent epithelial cells. Chemical cues from infected microbiome, dead cells, and immune cells, as well as physical cues from the empty space, initiate the migration of epithelial cells near the wound area within a few hours after injury (Ben Amar and Wu, 2014; Pastar et al., 2014; Chester and Brown, 2017; Krzyszczyk et al., 2018). The contribution of the epidermal basal and suprabasal layers has been long debated. Recently, intravital imaging revealed that both basal and suprabasal cells actively migrate with dynamic lamellipodial movements (Park et al., 2017). Migration tracking analysis identified that the speed of migration is vertically “gradient.” The bottom basal layer cells are fast, and middle spinous layer cells are slow. The top granular layer cells do not move, which could be due to the solid cell–cell adhesion *via* tight junctions. The participation of differentiated cells is crucial because inhibiting the migration of suprabasal cells delays wound closure dramatically (Park et al., 2017). This could be because of the lateral movement of the migrating cells, as well as because of the urgency to reduce the exposure to the external environment. During the re-epithelialization, proliferation is dramatically increased like the development after the initiation of migration to supply cells to the wound. Previously, it was considered that proliferation cannot occur in migrating cells. However, the proliferation and migration territories are partially overlapped, and cells in the overlapped territory, also known as a mixed zone, can perform both proliferation and migration at the same time. In addition, cells in the suprabasal layers can proliferate during wound repair similar to epidermal development (Richardson et al., 2014; Park et al., 2017; Damen et al., 2021). It remains unclear whether undifferentiated cells can be found in the upper layers because of premature delamination or because differentiated cells can dedifferentiate during wound repair. Finally, the last phase of the wound repair is remodeling. Since differentiation was increased during re-epithelialization, the epidermis is thicker than the normal epidermis before injury (Aragona et al., 2017; Park et al., 2017). The thick epidermis on the wounded area, also known as a neo-epidermis, slowly becomes thinner overtime during the remodeling. It is still unclear how the epidermal thickness is regulated and whether remodeling dermis impacts the epidermal thickness. Since apoptosis plays a crucial role to maintain epidermal homeostasis and to remodel psoriatic epidermis, increased apoptosis might be involved in thickness reduction (Weatherhead et al., 2011; Riwaldt et al., 2021). Alternatively, an enhanced differentiation rate could lead to faster shedding of cells from the skin’s surface, reminiscent of the periderm elimination during the development (Figure 2).

Several signaling factors play important roles in the development and in the wound repair. For example, calcium signaling and reactive oxygen species (ROS) are essential for the differentiation of the epidermis during development (Hamanaka et al., 2013; Bikle et al., 2014). These factors regulate collective migration and enhance proliferation of epithelial cells during wound repair (Hoffmann and Griffiths, 2018; Tu et al., 2019). In terms of the epidermal growth factor receptor (EGFR) signaling, EGFR KO mice the show abnormal epidermal structure during the development (Mascia et al., 2013). However, the EGFR is related to the migration and the proliferation of epithelial cells during wound repair, rather than the structural abnormalities (Repertinger et al., 2004). Collectively, these data suggest that signaling factors impact both the development and the wound repair, but regulatory mechanisms can be different depending on the circumstances of the tissue.

### Tissue-Resident Immune Cells in Development vs. Injury

During development, LCs are derived from two different sources. The first wave of precursors comes from the yolk sac, and the second wave of precursors comes from the fetal liver (Hoeffel et al., 2012). Since the yolk sac–derived LC precursors are mostly replaced by LC precursors from fetal liver, the latter is predominant in the adult epidermis (Hoeffel et al., 2012). The LC precursors in the fetal skin do not show dendritic morphology, and their number is much lower than LCs in the adult skin. The proliferation of LCs is dramatically increased, and LCs become mature within 1–2 weeks after birth (Doebel et al., 2017). Morphological and functional maturation, together with increased cell numbers, establish the regular immune network to create the immunological barrier (Deckers et al., 2018). Recently, single-cell RNA-seq and mass cytometry data revealed that there are two subsets of LCs, which are phenotypically and functionally distinct, in the human skin (Liu et al., 2021). However, it is unclear whether these subsets are dependent on their fetal origins.

Although mature LCs in the adult skin are fully differentiated, they can self-renew within the tissue much like tissue-resident macrophages (Doebel et al., 2017). Multicolor fate mapping demonstrated that mature LCs maintain their network within the epidermis without replenishment from precursors in the steady state (Ghigo et al., 2013). Therefore, about 5% of total LCs proliferate during homeostasis (Chorro et al., 2009). However, once immunological damage occurs, such as LC depletion, by using genetic mouse models with diphtheria toxin, UV irradiation, or inflammation, outer precursor cells repopulate the damaged area and differentiate to mature LCs (Kaplan, 2017). Similar to the developmental LC seeding, two distinct types of precursors repopulate at different time points (Seré et al., 2012). The first wave is from Gr-1^hi^ monocytes 1–2 weeks after damage, and LCs from these monocytes stay short term in the epidermis. The second wave is for long-term repopulation by bone marrow–derived precursors a few weeks after damage. LCs also recover their network after the skin injury. Previous studies showed that LCs are located on the neo-epidermis within 2–5 days. However, it is still unclear whether LCs repopulate during or after re-epithelialization like the development. Given the short time of repopulation, there is a possibility that LCs in the epidermis migrate together with epithelial cells during the re-epithelialization.

DETCs originate only from the fetal thymus during development (Havran and Jameson, 2010). Precursor cells maturate into DETCs in the epidermis and can self-renew in the adult skin during homeostasis like LCs (Gentek et al., 2018). However, in contrast to LCs, there are no additional precursors for replenishment in adults. Therefore, DETCs are not found in the damaged area after wound repair, but CD8^+^ T_RM_ covers the region instead (Zaid et al., 2014; Sandrock et al., 2018). Although DETCs do not participate in the reconstitution of the immunological barrier, activated DETCs near the wound edge directly contribute to re-epithelialization by enhancing proliferation with the secretion of keratinocyte growth factors (Jameson et al., 2002). Mobility and long-term fate of activated DETCs within the repairing epithelium need to be identified in the future.

## Conclusion

The epidermis is composed of diverse cell types, and each cell type has a specialized barrier function to protect our body. The proper cellular function can be exerted within the spatially organized structure. Wound repair is the process of restoring the organization in an orderly manner to regain pre-existing architecture. The epidermal architecture is initially built during embryonic development. Therefore, wound repair and embryonic development share a common goal of building/rebuilding a functional tissue and several common features of processes. Although the damaged skin is built well *via* the repairing process, it still has limitations compared to developmental skin formation such as scarring and loss of appendages. Comprehensive understanding of tissue creation by development and by the repair processes will provide new therapeutic strategies for more efficient healing.
